# Silica-silk fibroin hybrid (bio)aerogels: two-step versus one-step hybridization

**DOI:** 10.1007/s10971-019-04933-4

**Published:** 2019-02-28

**Authors:** Hajar Maleki, Nicola Huesing

**Affiliations:** 1 Chemistry and Physics of Materials, Paris-Lodron University Salzburg, Jakob-Haringer-Strasse 2a, 5020 Salzburg, Austria; 2 Institute of Inorganic Chemistry, Department of Chemistry, University of Cologne, Greinstraße, 6 50939 Cologne, Germany

**Keywords:** Aerogel, Silk fibroin, Hybrid, Mechanical reinforcement, Two step sol-gel, One step sol-gel

## Abstract

In this study, silk fibroin as a highly promising naturally occurring biopolymer extracted from silkworm cocoon is applied to mechanically reinforce silica aerogels. To this aim, two different approaches for the incorporation of silk fibroin into the silica network are compared: (1) a one-step acid catalyzed and (2) a two-step acid-base catalyzed sol–gel reaction. The total organosilane concentration, as well as the SF to silane mass fractions, regulated the hybridization process to proceed either through a one-step or two-step sol–gel reaction. In both processes, for an efficient chemical mixing the silk fibroin components with the silane phase, a silane coupling agent, 5-(trimethoxysilyl) pentanoic acid (TMSPA), comprising carboxylic acid groups and a pentyl hydrocarbon chain has been used. For a low organosilane content (3.4 mmol) along with a high SF to silane mass ratio (15–30%), the gelation of the silane and silk fibroin phases took place in a one-pot/one-step process in the presence of an acid catalyst in an entirely aqueous system. In the two-step synthesis approach, which was applied for high initial silane contents (17 mmol), and low SF to silane mass ratios (1–4%), first, the gelation of the silk fibroin phase was triggered by addition of an acid catalyst followed by a more pronounced condensation of the silane catalyzed by the addition of the base. Both synthesis approaches led to materials with promising mechanical properties—being 1) the one-step process resulting in gels with much better compressibility (up to 70% of strain), low density (0.17–0.22 g cm^−3^) and three orders of magnitude improvement in the Young’s modulus (13.5 MPa) compared to that of the pristine silica aerogel but with rather high shrinkage (30–40%). The two-step process in principle could result in the hybrid aerogel with interesting bulk density (0.17–0.28 g cm^−3^) with lower shrinkage (10%), but the resultant aerogel was stiff and fragile. Also, both approaches led to a significant reduction in the time required to prepare strong hybrid aerogels compared to conventional hybrid polymer-silica aerogels with the utilization of an entirely aqueous synthesis approach for a wide range of applications.

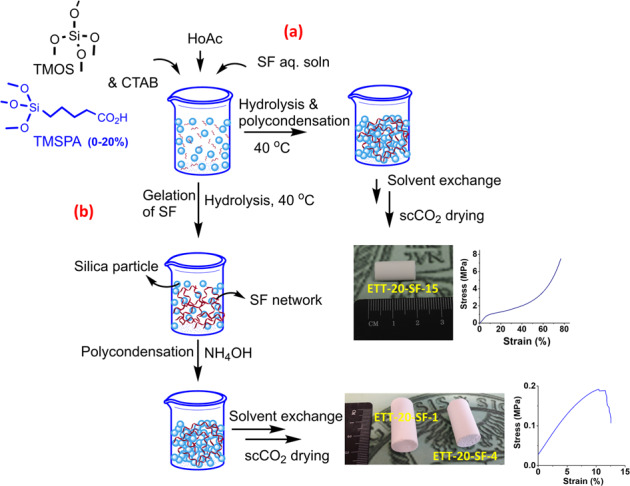

## Highlights

- Silk fibroin (SF) biopolymer is an interesting biopolymer for mechanical reinforcement of silica aerogel.
- Two sustainable sol–gel based approaches are proposed for hybridization of silica with SF.
- Hybrids obtained by the one step sol–gel synthesis approach demonstrate better mechanical performance.
- Molar ratio of SF to organosilanes is the most determinant factor for resulting final properties.
- Both synthesis approaches led to significant reduction in the time required for preparation of strong hybrid aerogels.

## Introduction

Highly porous and lightweight silica-based aerogels are of interest for a multitude of high-performance applications, such as thermal insulation in the construction, building and aerospace sector, environmental cleaning, biomedical, and pharmaceutical applications to name only a few [1]. However, despite their peculiar physical properties, they often show inferior mechanical characteristics that limit their applicability. In recent years, different approaches have been proposed to improve the mechanical strength of silica and silsesquioxane based aerogels, e.g., (a) by using ORganically MOdified SILanes, *ORMOSILs*, having non-hydrolyzable moieties in the network in order to replace the short siloxane (Si–O–Si) bonds with more flexible -Si-carbon chains, (b) by polymerization or crosslinking the silica network with flexible organic monomers/polymers, and (c) by using various fiber networks (i.e., carbon, ceramic and polymeric fibers) [2]. Amongst these approaches, the polymerization/cross-linking of the silica network skeleton with an appropriate organic monomer or cross-linker is counted as an elegant mechanical reinforcement method [3, 4]. Crosslinking of the silica network with polymerizable organic monomers in most cases leads to promising results in terms of mechanical properties. However, in this approach tedious multi-step processes are required in which first hydrolysis and polycondensation of the silane phase is initiated, followed by polymerization/crosslinking of the organic monomer inside the silica and/or silsesquioxane network or the other way round [2].

In many cases, this requires more time along with the necessity for the extensive use of (non-green) organic solvents during the synthesis/polymerization and post-synthesis processing [2]. These shortcomings, however, have been solved by replacing these oil-based polymers with green biopolymers from various natural, renewable resources, such as different polysaccharide-based polymers, e.g., bacterial and plant-based cellulose [5]. One promising example in this regard is the work published by Cai et al. [6] in which the in-situ incorporation/gelation of tetraalkoxysilanes inside a cellulose-based fiber network conferred a very good mechanical resiliency to the resulting hybrid gels.

Applying natural biopolymers has several advantages, with the most important ones being: (1) in most cases it leads to a reduction of the time required for the synthesis of the hybrid gels, and (2) purely aqueous processing is possible and (c) by carefully controlling the rates of hydrolysis and condensation, a homogeneously mixed hybrid is obtained by simultaneously initiating the sol–gel transition of the biopolymer and silane phase [7–9].

Silk fibroin (SF), a protein-based biopolymer extracted from *B. mori* silkworm cocoon has recently been utilized in the development of various functional materials, as this biopolymer has several assets in terms of biocompatibility, versatility in the processing into various shapes combined with a well-studied chemistry [10]. The concept of preparation of silk fibroin as aerogels and its utilization to mechanically support the delicate structure of silica and silsesquioxane aerogels is a new trend and has only recently been reported by our group [11, 12]. In this regards, ultra-light silk fibroin aerogels (so-called *AeroSF*) (*ρ*_b_ = 0.02 g cm^−3^) have been synthesized. Potential applications can be found, e.g., as thermal insulator material due to its very low thermal conductivity (0.028 W/mK). Also, superhydrophobic/ oleophilic PMSQ-silk fibroin hybrid aerogels have been developed which can be applied as a filter for the continuous separation of oil from water/oil mixtures. Additive manufacturing approaches, such as 3D printing, have been successfully utilized to prepare silica-silk fibroin aerogels with a deliberately adjusted shape and morphology, thus opening new technical fields, e.g., as multi-pore biomaterials or customized anisotropic thermal insulators. For all hybrid systems, a carboxylic acid functionalized coupling agent, 5-(trimethoxysilyl) pentanoic acid (TMSPA), allowed a homogeneous chemical mixing of both phases at the molecular scale [11, 12]. Hybridization of the silk fibroin with simple tetrafunctional silanes in the presence of an acid catalyst led to homogeneous mixing of both phases in a simple single step processing without any significant phase separation. However, in all established procedures that either rely on tri-functional organosilanes like methyltrimethoxysilane (MTMS) or tetrafunctional silanes in higher concentration very slow kinetics of the sol–gel transition are observed. Therefore, for both cases the sol–gel reaction usually requires an additional, base-catalyzed step to promote the polycondensation of the silane phase to result in a homogeneous, stable gel.

**Fig. 1:**
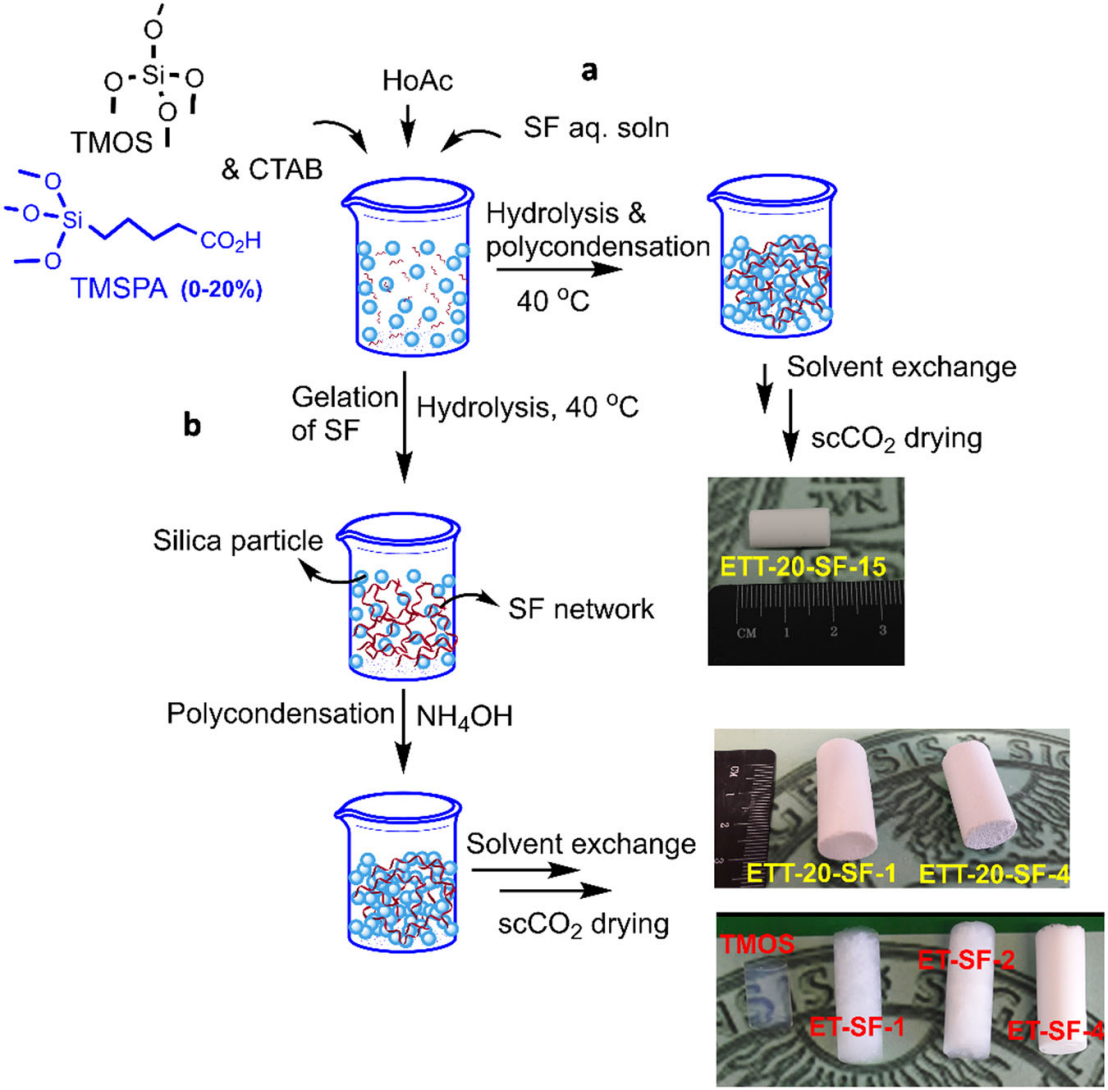
Schematic of the two synthesis methods for the preparation of silica-SF aerogel hybrids from the extracted SF solution in the presence of TMOS and TMSPA, through **a** one-step and **b** two-step sol–gel reaction followed by SCD with a photograph of representative cylindrical monoliths, from each synthesis approach

In a pH range close to the isoelectric point of silk fibroin (PI = 3.8–3.9), a stable gel can be formed. In contrast, at pH < 1.5 as well as pH > 13 silk fibroin is reluctant to gel [13]. To induce gel formation of the silane phase at pH values below 4, thus aiming at a concurrent gelation with the SF phase, a certain concentration of the silane is needed as at high silane concentration, the silane phase would require the addition of a base catalyst to turn to a gel state. Therefore, in order to adapt the gelation of the silane phase to the gelation kinetics of the silk fibroin, a careful control of the gelation kinetics of the process by regulating the total organosilane and acid concentration, SF mass ratio with respect to the silane and base catalyst concentration are needed. In this work, with considering all the facts given above, we aim to synthesize a series of homogeneous silica-silk fibroin hybrid aerogels by applying two different synthesis approaches of either a one-step acid catalyzed sol-gel approach, when a low silane concetration, (3.4 mmol) is used or a two-step acid-base catalyzed reaction (see Fig. 1), when a high content of the silane (17 mmol) is used. With both synthesis methods, the final goal is to investigate how the silane and SF contents influence the resulting materials structural properties as well as the derived mechanical performance.

## Methods

### Materials

*B. Mori* silkworm cocoons were purchased from Wild Fibers, UK. Tetramethyl orthosilicate (98% purity, TMOS), hexadecyltrimethylammonium bromide (98% purity, CTAB), methanol (99.8%, MeOH) and trimethoxysilane (95% purity), 4-pentanoic acid, (≥98% purity), lithium bromide anhydrous, (99.99% purity, LiBr), ammonium hydroxide (28–30%, NH_4_OH), sodium carbonate (Na_2_CO_3_), were procured from Sigma Aldrich. Slide-A-Lyzer™ G2 dialysis cassettes, (3.5 K MWCO, 3–5 mL) were purchased from ThermoFisher. All chemicals were used without further purification. Slide-A-Lyzer™ Dialysis Cassettes with molecular weight cutoffs (MWCOs) of 3.5 kD and volume capacity of 3.5–5 mL was purchased from ThermFisher SCIENTIFIC.

### Silk fibroin extraction

The SF aqueous solution was extracted from silkworm cocoon through a slightly modified standard procedure reported in Nature Protocols by Kaplan et al. [10]. First, silk cocoons (5 g) were cut into dime-sized pieces and boiled for 30 min in 2 L of aqueous Na_2_CO_3_ (0.02 M). The resulting fibers were rinsed with plenty of ultrapure water and dried overnight. The dried silk fibers are dissolved in LiBr (aq) (12–15 M) solution at 60 °C for 4 h and were then dialyzed against ultra-pure water for 48 h. The dialyzed SF solution is centrifuged twice at 9000 rpm and the supernatant is stored at 4 °C for later use.

### Synthesis of silica-SF aerogel hybrids

TMSPA is synthesized following a procedure previously published by our group [14]. To obtain silica-SF composites a two-step acid-base and a one-step acid catalyzed sol–gel reaction is performed. In both approaches, the hybrid samples are denominated as ET-SF-*y* (samples without addition of coupling agent, and ET refers to **E**xperiments with only **T**MOS) and ETT-*x-*SF-*y* (samples with addition of coupling agent, and ETT refers to **E**xperiments with **T**MOS and **T**MSPA), where “*x*” and “*y*” represent the percentage of TMSPA and SF, respectively. In the one-step acid-catalyzed sol–gel reaction, (*cf*. Fig. 1a) a total silicon molar content [Si] of 3.4 mmol is used while in the two-step catalyzed approach (*cf*. Fig. 1b), the total molar silicon content [Si] is 17 mmol. The sol in both systems comprises (organo)silanes (TMOS and TMSPA), SF, CTAB, and acetic acid. The TMSPA concentration is varied from 0 to 20 mol% of the total amount of silicon centers. The SF biopolymer concentration has been adjusted to 15 or 30 wt/vol% and 1 or 4 wt/vol% with respect to the total amount of silicon, for the one-step and two-step reactions, respectively. The aqueous acetic acidic solvent for the one-step sol–gel reaction varied for 100 and 120 mM for a system with 15 and 30 wt/vol% of SF, respectively. In the two-step reaction, first, the gelation of SF, as well as the hydrolysis of the organosilanes, occurred in 10 mM acetic acid, followed by the slow addition of the base catalyst (NH_4_OH, 1 mL, 2.8%). Phase separation was suppressed in both approaches by the addition of a surfactant, hexadecyltrimethlyammonium bromide (CTAB, 0.25 g). Depending on the TMSPA concentration, the overall gelation time varied from ~1 to 10 h for ET-x-SF-y and ETT-x-SF-y, respectively. The homogeneous hybrid silica-SF gels were aged in an oven (40 °C, 2 days). By-products were extracted by solvent exchange to methanol, followed by drying of the filigree wet gels with supercritical CO_2_.

### Characterization techniques

The bulk density was calculated from the mass and the volume of the cylindrical aerogel samples. The skeletal density was measured using a helium pycnometer (AccuPyc II 1340, Micromeritics, USA). TEM images were recorded with a JEOL 200F cold field-emission (W) filament, 200 keV operated at an accelerating voltage of 300 kV. EDS-TEM elemental mappings were recorded by a JEOL, Centurio 100, detector area of 100 mm, the solid angle of 0.97 sr, and resolution of ~133 eV for Mn. Scanning electron microscopy (SEM) images were taken with a scanning electron microscope (Zeiss ULTRA Plus) running at 5–10 kV with an in-lens detector and a working distance of 3 mm. Attenuated total reflection Fourier transform infrared spectra (ATR-FTIR) were obtained on a Bruker Vertex 70 spectrometer with a 4 cm^−1^ resolution spectrophotometer (scan range 400–4000 cm^−1^), and the spectra were plotted in the range of 1400–2000 cm^−1^. Nitrogen adsorption-desorption measurements were carried out at 77 K using a Micrometrics ASAP 2420. Before the analysis, the sample was outgassed at 60 °C in vacuum (10^−5^ bar) for 24 h, to remove adsorbed species. The specific surface area was calculated with the Brunauer, Emmett and Teller 5-point method in the relative pressure range of 0.05–0.3. From the skeleton and bulk densities values, the porosity *ε* (%), pore volume (*V*_p_) and pore diameter (*D*_p_) of the samples were calculated.

Mechanical characterization of the composites was carried out on monolithic cylindrical samples using a universal mechanical testing equipment (Zwick/Z010, Zwick/Roell, Germany), equipped with a 1 kN force transducer (KAP-S, AST Gruppe GmbH, Germany) in a controlled environment (23 °C, 50% relative humidity). Stress-strain curves were plotted in compression mode, and elastic moduli were calculated from the linear range of the curves, which typically occurred at 3 ± 1% strain. A constant deformation rate of 0.5 mm/min was used, and final strength was taken at the first signs of buckling which occurred typically at 20–70% strain.

## Results and discussion

The detailed formulations along with the key properties of the synthesized silica-silk fibroin aerogels have been summarized in Table 1. In this work, we present two synthesis approaches: a one-step procedure, in which hybridization of silk fibroin and the silica network is performed in a one-pot sol–gel reaction of silk fibroin with a small amount of the silanes, tetramethoxysilane and TMSPA, (3.4 mmol), and a high mass fraction of SF to silane (SF: Si, 15:100 and 30:100) in the presence of an acid catalyst. The second approach involves a two-step sol–gel reaction with a high silane concentration (17 mmol) and a small mass fraction of SF to silane (SF: Si, 1:100 and 4:100) in which the gelation of the SF biopolymer in the aqueous sol is initiated by an acid catalyst with an increase on the sol viscosity (in 15 min) followed by the addition of a base to trigger polycondensation and/or gelation of the silane phase. In fact, in the two-step reaction, a high silane concentration in the sol mixture required an extra step of the addition of a base catalyst in order to start the polycondensation and gelation in the silane phase. This has been done by control of the gelation kinetics of SF by the acid catalyst concentration followed by the polycondensation and gelation of the hydrolyzed organosilane species in the presence of a base catalyst. The gelation occurred immediately after addition of the base. Also, as it is evident from the photographs of the different samples shown in Fig. 1b, a successive increase in the SF content results in an increasing opaqueness due to phase separation effects. For the one-step sol–gel process, the reaction occurred entirely in one-step, although the gelation time has significantly increased from several minutes to several hours and the final material typically undergoes a relatively higher shrinkage.

**Table 1:** Physical properties of silica-SF hybrid aerogels synthesized via the two different synthesis approaches

Aerogel	*ρ*_bulk_ (g cm^−3^)	*ρ*_skeleton_ (g cm^−3^)	Dimensional shrinkage (%)	*ε* (%)	*S*_BET_ (m^2^ g^−1^)	*V*_pore_ (cm^3^ g^−1^)	D_pore_ (nm)	Compressive strength, *δ*_max,_ (MPa)	*E* modulus (MPa)
*Two-step acid-base catalyzed sol–gel reaction*									
ET-SF-1	0.177 ± 0.03	2.12	8	92	1011	5.17	20.45	0.46	6.84
ET-SF-4	0.183 ± 0.02	2.15	11	91	610	4.99	32.7	0.24	3.30
ETT-20-SF-1	0.230 ± 0.03	1.95	17	88	433	3.83	35.3	0.18	6.18
ETT-20-SF-4	0.289 ± 0.03	1.72	18	83	260	2.87	44.1	0.74	4.59
*One-step* (in situ) *acid catalyzed sol–gel reaction*									
ET-SF-15	0.172 ± 0.03	2.04	28	91	798	5.16	37.0	2.17	9.27
ETT-20-SF-15	0.220 ± 0.02	1.83	40	88	353	3.99	60.0	7.45	13.55
ETT-20-SF-30	0.180 ± 0.03	1.82	36	90	332	5.00	79.0	1.19	6.40

Bulk (*ρ*_bulk_) and skeletal (*ρ*_skeleton_) density, porosity(ε%,$\frac{{1/\rho _{\mathrm{b}} - 1/\rho _{\mathrm{s}}}}{{1/\rho _{\mathrm{b}}}} \times 100$), specific surface area (*S*_BET_), pore volume (*V*_pore_,$1/\rho _{\mathrm{b}} - 1/\rho_{\mathrm{s}}$), pore diameter (*D*_pore_, $4V_{\mathrm{p}}/S_{{\mathrm{BET}}}$)) of the silica-SF aerogel hybrids

**Fig. 2:**
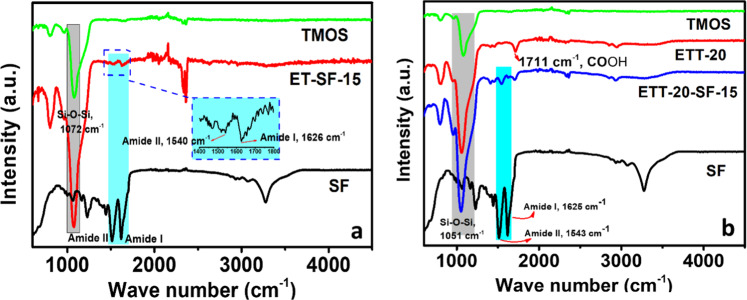
ATR-FTIR spectra of reference tetramethoxysilane-based silica (TMOS) and silk fibroin gels (SF) and representative silica-SF aerogels **a** without coupling agent **b** with a coupling agent

In both approaches, a coupling agent containing a carboxylic acid functionality, 5-(trimethoxysilyl) pentanoic acid (TMSPA), is co-condensed to give a homogeneous inorganic–organic hybrid silica network. This coupling agent is added to the sol–gel system for two reasons: on one hand, its long hydrocarbon chain increases the flexibility and reduces the fragility of the silica network and, on the other, the carboxylic acid moiety results in stronger interactions between both phases by various possible covalent and non-covalent interaction scenarios [11]. In addition, the acidic character of the coupling agent assists the concomitant hydrolysis and polycondensation of the alkoxysilanes with the physical gelation of the SF phase. ATR-FTIR spectra indicate the successful modification of the silica network with carboxylic acid functionalities (see, i.e., the spectrum of ETT-20 in Fig. 2b) by the presence of characteristic stretching vibrations of the carboxy groups (–C(=O)OH) at a frequency of ~1711 cm^−1^. Also, the incorporation of the silk fibroin into the silica network (see the spectra of ETT-20-SF-4 and ET-SF-4 in Fig. 2a, b, respectively) is strongly supported by the presence of amide I (*ν*_as_ (C=O): 1626 cm^−1^) and amide II (*δ*_s_ (N–H) deformation/bending: ~1540 cm^−1^) vibrations [15]. For these samples, the characteristic band of the carboxy group at 1711 cm^−1^ (see spectrum ETT-20) is shifted to wave number of 1698 cm^−1^ (as found for ETT-20-SF-15), supporting the involvement of the carboxylic acid functionality in the interactions with SF through, e.g., amidic bonds.

Despite the higher dimensional shrinkage (*cf*. Table 1) for the monoliths processed with one-step acid catalyzed gelation, the average densities are still relatively low compared to those prepared by the two-step acid-base-catalyzed process. For both systems, an increase of the overall mass of the monoliths imposed by incorporation of massive hydrocarbon chain of TMSPA as well as loaded SF biopolymers resulted in an increase of the bulk densities. For the one-step process, especially for those samples comprising 20 mol% of TMSPA, the density has also been influenced by the rather high dimensional shrinkage (40%) upon synthesis and processing. However, despite the fact given above, the density for ETT-20-SF-30 is rather low (*ρ*_b_ = 0.18 g cm^−3^) compared to that of sample ETT-20-SF-15 (*ρ*_b_ = 0.22 g cm^−3^), which can be explained by the steric hindrance of the functional groups of both phases due to the high concentration of surface functionalities and SF. Based on the previous study [11], with quantifying the overall organic moieties of the samples within the monolith by the thermogravimetric studies, a considerably higher organic contribution/ mass fraction of SF in case of ETT-20-SF-15 was also observed.

**Fig. 3:**
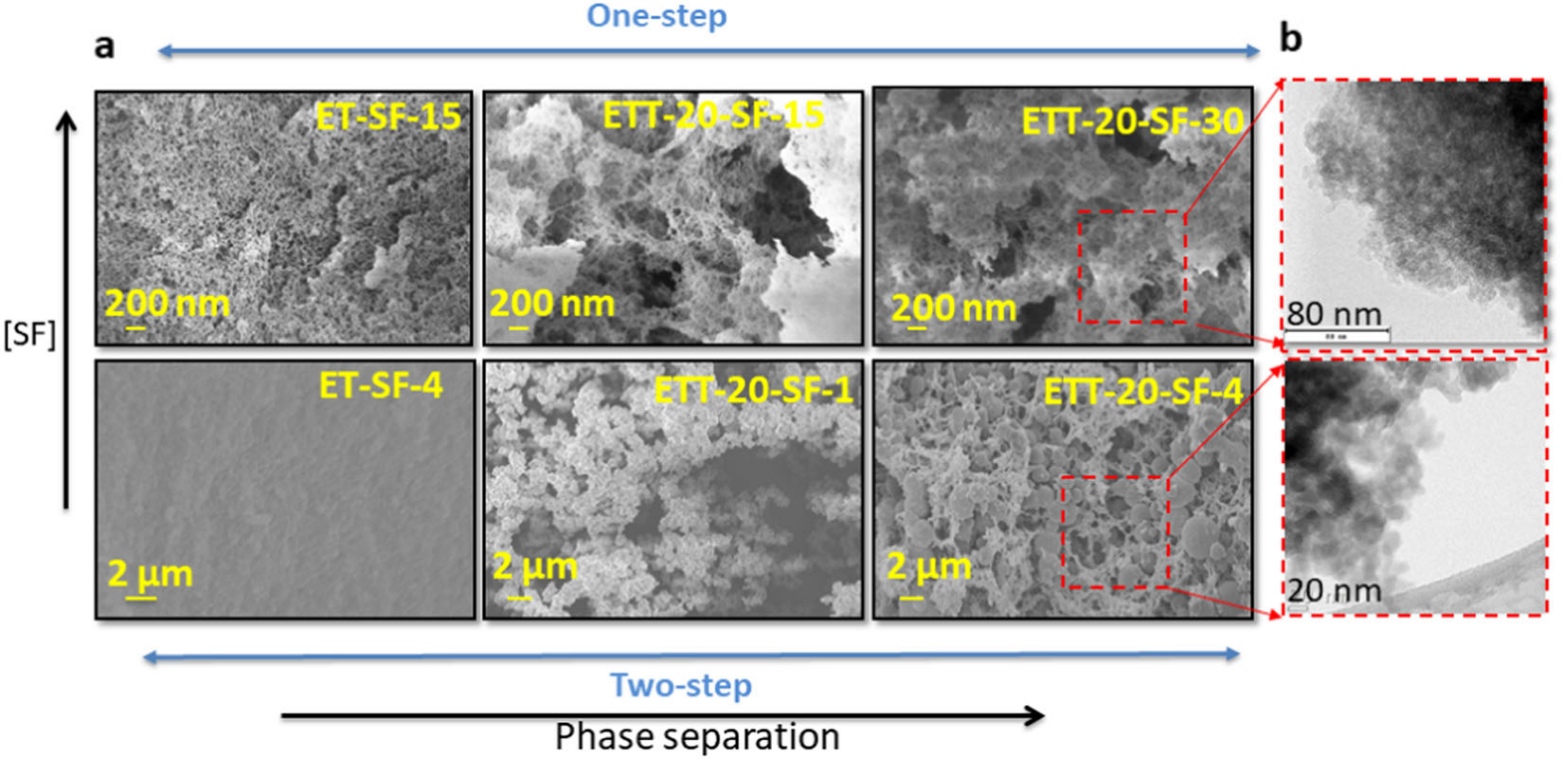
**a** SEM and **b** TEM micrographs of some representative silica-SF aerogel hybrids obtained by one-step acid catalyzed and two-step acid-base catalyzed sol–gel reaction with increasing the SF and TMSPA contents

**Fig. 4:**
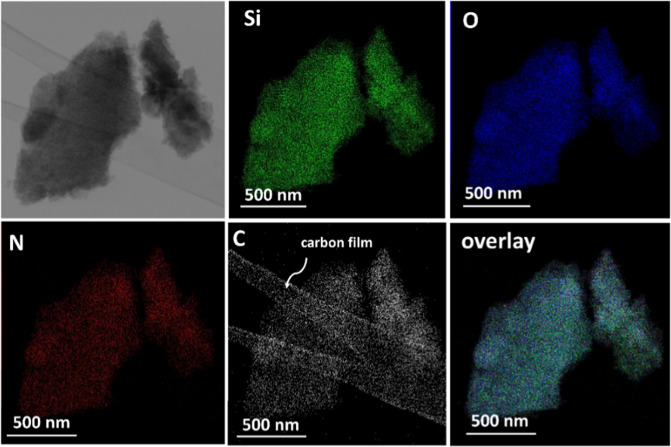
EDS-TEM elemental mapping of ET-SF-30 aerogel hybrid

The SEM and TEM micrographs of representative silica-SF aerogels are shown in Fig. 3a, b. The network of the hybrid aerogels prepared by the one-step acid catalyzed sol-gel reaction is built-up by a bicontinuous fibrous structure in which increasing contents of both, SF and TMSPA, cause phase separation resulting in open porous structures with micron-sized pores in the monoliths. In the two-step sol–gel reaction, a globular hybrid structure is found in which the addition of TMSPA plays a significant role in the development of the macroporosity via phase separation concomitant with a coarsening of the network forming particles. The TEM micrograph of ETT-20-SF-30 indicates a polymer-like interpenetrated network of silica and SF phases with small pores. This is expected for a gel obtained by an acid catalyzed sol–gel reaction having a cluster–cluster growth mechanism. ETT-20-SF-4 is a particulate gel with an interpenetrated network structure of rather big colloidal particles associated with its monomer—cluster growth model for the network build-up [16, 17]. Moreover, as it is evident from the elemental mapping of the individual elements by Energy dispersive X-ray spectrometry (EDS)-TEM, Fig. 4, all the essential elements belonging to both phases are homogeneously distributed in the studied regions, thus confirming a homogeneous mixing of both silica and SF phases.

For both synthesis strategies, the specific surface area and pore volumes of the hybrid monoliths decreased by an increase in the silk fibroin concentration.

**Fig. 5:**
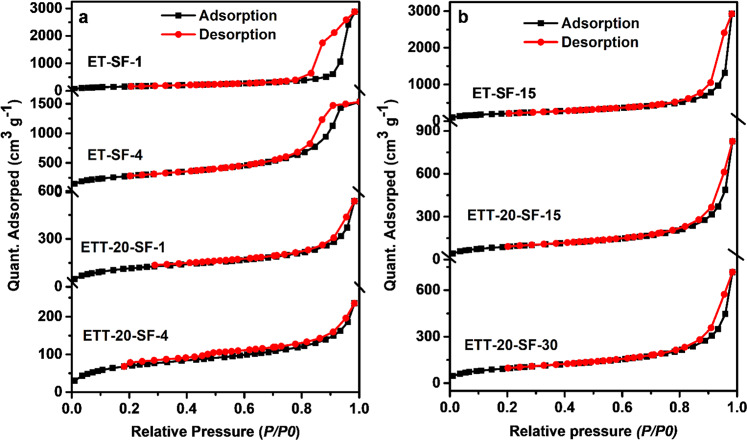
N_2_ adsorption–desorption of developed silica-SF hybrid aerogels obtained by two **a** and one-step sol–gel reactions **b**

The N_2_ adsorption–desorption isotherms (Fig. 5) indicate that all hybrid aerogels have a mesoporous structure evidenced by the type IV isotherm with capillary condensation occurring at *p*/*p*_0_ > 0.4. In samples obtained by the one-step gelation (ET-SF-15, ETT-20-SF-15, and ETT-20-SF-30), no changes in the hysteresis loop occur which is indicative of a lower mechanical deformation of the samples upon desorption of nitrogen that has been condensed in the capillaries, therefore confirming the stiffness of these hybrid materials compared to the two-step sol–gel reaction. Upon addition of SF and TMSPA to the TMOS-based monoliths, the average pore diameters (*cf*. Table 1) in all hybrid monolith increase.

**Fig. 6:**
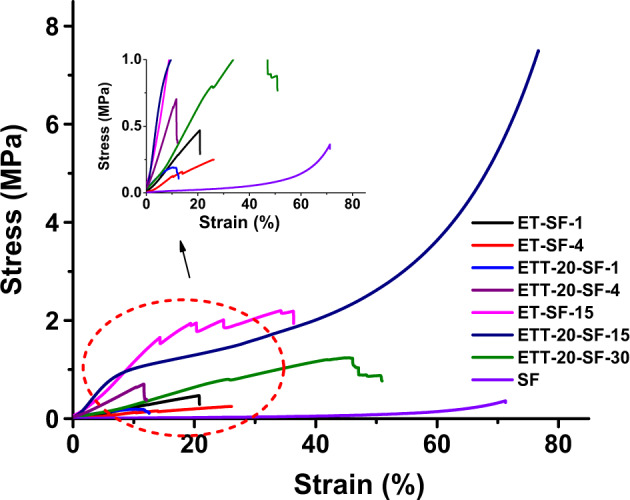
Stress–strain curves of representative silica-SF aerogel hybrids obtained with both, one-step and two-step, sol–gel reactions

The mechanical performance of the silica-SF hybrid aerogels was accessed by uniaxial compression tests. As it is evident from the stress-strain curves (Fig. 6), the compressibility, as well as the maximum compressive strength that is tolerated by the monoliths without structural failure, depends on the SF content in relation to the amount of silane, as well as the presence of coupling agents in the monolith. Compared to pure SF aerogels—*AeroSF* [11] (obtained by one-step acid catalyzed reaction), which is compressible up to 70% of strain, the aerogel hybrids obtained by the two-step sol–gel reaction show a lower compressibility (e.g., compressible up to 45% of strain). This can easily be explained by the higher inorganic content in these gels. Instead, the samples from the one-step sol–gel reaction with the same density contain a high viscoelasticity and compressibility compared to two-step sol–gel reaction which originating from the flexible hydrocarbon chains of the SF biopolymer due the a high contribution of SF polymer in the network formation as well as the elastic siloxane network in the skeleton upon incorporation of flexible pentyl chain in TMSPA, which render the aerogel monolith with a high deformability without fracture of the skeleton on compression. In general, the samples obtained by the one-step sol–gel reaction are tougher (e.g., larger area under the stress–strain curve), stronger (higher maximum strength, *δ*_max,_ = 7.45, and Young’s modulus, *ε* = 13 MPa) and more compressible (70% of strain, in case of ETT-20-SF-15) compared to those that are obtained by two-step sol–gel reaction.

## Conclusions

In this study, two simple synthesis routes—a two-step acid-base catalyzed and a one-step acid catalyzed sol–gel approach—to mechanically reinforce the delicate structure of silica aerogels using silk fibroin biopolymer are compared. In both approaches, the extracted silk fibroin biopolymer acts as a platform to mechanically support the delicate structure of the silica aerogel by means of a carboxylic acid functionalized organosilane as a coupling agent. The silanes content, the SF to silane mass fraction are the most determining factors for the later materials properties when conducting the reaction through a single step acid catalyzed or two-step acid-base catalyzed sol–gel process. At a low silane content, e.g., Si = 3.4 mmol or at a high mass fraction of SF, the gelation of both phases occurred in the presence of an acid catalyst in a single step, however, the rate of condensation, thus time for gelation, for a formulation containing coupling agents is very slow. For a high silane content, e.g., Si = 17 mmol, after gelation of the SF phase, the reaction requires a base catalyst to homogeneously polycondense the silane phase within the pre-formed SF network. In both approaches, an interpenetrated network of silica-SF has been formed in which both components are homogeneously distributed at a molecular scale. The silica-SF aerogel hybrids obtained by the one-step synthesis approach are mechanically stronger compared to the samples from the two-step procedure. They also demonstrate high flexibility originating from the flexible hydrocarbon chain of the coupling agent as well as the resilience of silk fibroin biopolymer chains within the aerogel network. Finally, the mechanically strong and lightweight hybrid silica-SF aerogels prepared in this study are expected to have a high impact for further load bearing applications mainly for thermal insulation.
